# Raynaud's Disease I

**Published:** 1908-11-28

**Authors:** 


### RAYNAUD'S DISEASE I.

The condition to which the term Raynaud's disease is applied is a neurosis, and it is perhaps one of the most useful examples that can be brought to illustrate the fact that a neurosis is a disease. A neurosis is a disease of function without disease of structure, as distinct from organic lesions which are diseases of structure with more or less secondary functional disablement.

A neurosis is just as real a disease as is an organic affection, but in one sense it is, of course, far less serious—it seldom causes death. In many functional diseases or neuroses there are no objective signs at all; the symptoms are all subjective and the words used by the patient in describing them are sometimes of the most extravagant and apparently unreal; in such cases the whole disease is apt to be treated with a shrug of the shoulders, actual or concealed. It is difficult to realise the symptoms when they are thus subjective, but when they are objective the fact of there being a very real disease is obvious to all, as in Raynaud's disease.

The nerves whose functions are being imperfectly or erroneously performed in this condition are the vaso-motor nerves, especially those of the fingers and hands, toes and feet; less frequently, those of the ears, nose, tongue, and face; and least often those of the trunk.

The vaso-motor nerves, instead of controlling the blood supply to the affected parts in an orderly and efficient manner, do so improperly as the result of comparatively trifling exciting causes. The changes which occur in the affected parts in consequence of this are usually described in three stages, namely: (1) local syncope, (2) local asphyxia, and (3) local death, or gangrene.

1. *Local syncope.*—If a person has been in the water bathing for a longer time than usual it is not uncommon for him to find when he gets out to dry himself that some or all of his fingers have gone deathly pallid. This pallidity is doubtless due in great part to deficient cardiac force, which is unable, in an untrained person, to maintain the distal circulation in full vigour against the combined effects of unusual exertion and unusual exposure to cold. It is sometimes said to be a sign of poor circulation. The same occurs in many people in cold weather.

When this occurrence of "dead men's fingers"—which is, in fact, local syncope—is due to an adequate cause it is not termed Raynaud's disease; but when it develops without adequate cause, and upon all sorts of occasions so as to be a nuisance to the patient, it is no longer a natural phenomenon, and it is the first symptom of Raynaud's disease. The most common exciting cause of the local syncope is exposure to cold, so that the attacks are commoner in winter than they are in summer; but they come on with so trifling an exposure that they constitute a diseased process. The latter consists in local spasm of the arterioles due to erroneous over-action of the vaso-constrictor nerves; and the circulation in the part may entirely cease for the time being.

The patients are nearly all of the female sex, and the disease starts, as a rule, between the ages of 15 and 25. Once begun it is extremely liable to persist throughout life, in spite of treatment; but it may remain in the stage of spasmodic attacks of local syncope without ever progressing into the second or third phase of the malady.

2. *Local asphyxia* may occur without antecedent local syncope; but more often local syncopal attacks occur first, and it is only later that fingers or toes which used merely to go dead white now become purple or almost black whenever the patient is seized by an attack. It is a later effect of vaso-motor spasm, due to dilatation of the minute veins, and to extreme venosity of the blood stagnating within them. The condition finds its exact analogy in the lividity of parts that have been unduly exposed to extreme cold, and like such parts, the tissues affected by the local asphyxia of the second stage of Raynaud's disease are excessively painful, particularly when the asphyxia is passing off and the circulation is beginning again in the parts in which it has been temporarily arrested.

3. *Local gangrene.*—It is not surprising that parts are less well-nourished and resistant than they should be when their vascular supply is so greatly interfered with as is the case in the fingers and toes of a patient who suffers attacks of local syncope or of local asphyxia in them on the slightest provocation.

The result of this condition is that comparatively slight injuries may cause extensive damage in parts affected by Raynaud's disease. Even apart from any known injury, sore places analogous to breaking-down chilblains are very apt to occur on the fingers and toes and other affected parts. These areas of local necrosis of the soft parts—local gangrene as it is called—readily heal when hot weather comes, their site being then marked by irregular scars and deformities.

It will be seen, therefore, that the phenomena of Raynaud's disease are precisely similar to the effects of increasing degrees of cold—dead men's fingers first, then lividity and painfulness of the previously benumbed parts, and in severe cases necrosis of the soft parts akin to frost-bite or to breaking-down chilblains.

The point, however, is that a patient afflicted with Raynaud's disease has vaso-motor nerves that are far too easily influenced by cold—spasmodic attacks of local syncope, local asphyxia, or local gangrene arising from what ought to be inadequate causes. There are certain other lesions that may be associated with Raynaud's disease. These will be briefly discussed when we come to give an account of the treatment of the disease.

(*To be continued.*)

